# Role of Retinoic Acid Signaling, FGF Signaling and *Meis* Genes in Control of Limb Development

**DOI:** 10.3390/biom11010080

**Published:** 2021-01-09

**Authors:** Marie Berenguer, Gregg Duester

**Affiliations:** Development, Aging, and Regeneration Program, Sanford Burnham Prebys Medical Discovery Institute, 10901 N. Torrey Pines Road, La Jolla, CA 92037, USA; mberenguer@sbpdiscovery.org

**Keywords:** retinoic acid, FGF, *Meis*, limb development

## Abstract

The function of retinoic acid (RA) during limb development is still debated, as loss and gain of function studies led to opposite conclusions. With regard to limb initiation, genetic studies demonstrated that activation of FGF10 signaling is required for the emergence of limb buds from the trunk, with *Tbx5* and RA signaling acting upstream in the forelimb field, whereas *Tbx4* and *Pitx1* act upstream in the hindlimb field. Early studies in chick embryos suggested that RA as well as *Meis1* and *Meis2* (*Meis1/2*) are required for subsequent proximodistal patterning of both forelimbs and hindlimbs, with RA diffusing from the trunk, functioning to activate *Meis1/2* specifically in the proximal limb bud mesoderm. However, genetic loss of RA signaling does not result in loss of limb *Meis1/2* expression and limb patterning is normal, although *Meis1/2* expression is reduced in trunk somitic mesoderm. More recent studies demonstrated that global genetic loss of *Meis1/2* results in a somite defect and failure of limb bud initiation. Other new studies reported that conditional genetic loss of *Meis1/2* in the limb results in proximodistal patterning defects, and distal FGF8 signaling represses *Meis1/2* to constrain its expression to the proximal limb. In this review, we hypothesize that RA and *Meis1/2* both function in the trunk to initiate forelimb bud initiation, but that limb *Meis1/2* expression is activated proximally by a factor other than RA and repressed distally by FGF8 to generate proximodistal patterning.

## 1. Introduction

Investigation of the mechanisms underlying limb development serves as a paradigm for understanding development in general. Many signaling and transcriptional pathways converge to generate growth and patterning of the limb [1]. Early studies found that the emergence of limb buds from the trunk is dependent upon fibroblast growth factor 10 (FGF10) signaling [2], whereas subsequent growth of the limb also requires FGF8 signaling [3,4]. During forelimb bud initiation, *Tbx5* is required upstream of FGF10 [5,6,7] and retinoic acid (RA) is required upstream of *Tbx5* [8]. During hindlimb bud initiation, *Tbx4* and *Pitx1* function upstream of FGF10 [9]. Subsequent proximodistal patterning of both forelimbs and hindlimbs was suggested from studies in chick embryos to require proximal-specific expression of *Meis1* and *Meis2* (*Meis1/2*) as well as RA signaling, which was proposed to activate *Meis1/2* expression proximally [10,11,12]. However, genetic loss of RA signaling in mouse embryos using knockout mutations of RA-generating enzymes encoded by *Aldh1a2* or *Rdh10* resulted in no change in proximal limb *Meis1/2* expression and normal limb patterning [8,13,14]. Here, we discuss the mechanisms of limb initiation and proximodistal patterning in the light of recent genetic studies on RA signaling and *Meis1/2* function.

## 2. Requirement of Retinoic Acid for Forelimb Bud Initiation

### 2.1. Mechanism of Retinoic Acid (RA) Signaling

**Figure 1:**
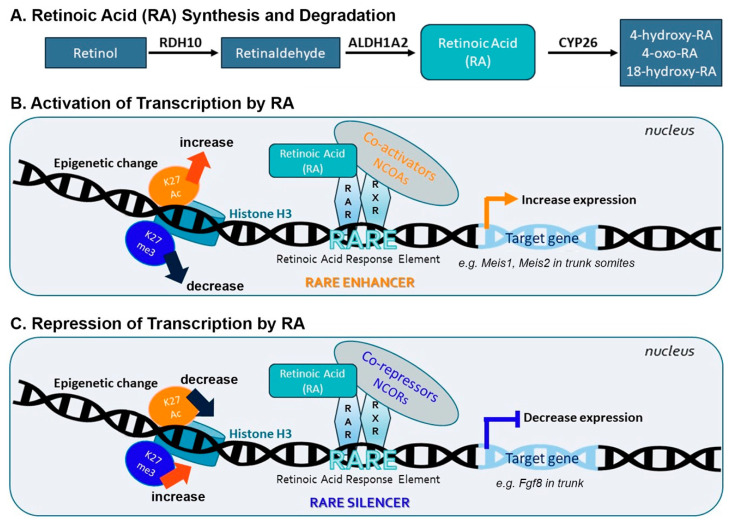
Generation of retinoic acid (RA) and molecular mechanism used by RA to control transcriptional activation or repression of target genes. (**A**) RA is synthesized by sequential conversion of retinol (vitamin A) to retinaldehyde by retinol dehydrogenase 10 (RDH10), followed by metabolism of retinaldehyde to RA. RA can be degraded by CYP26 enzymes. (**B**,**C**) The heterodimer formed by nuclear RA receptors (RARs) complexed with retinoid X receptors (RXRs) binds noncoding DNA sequences called RA response elements (RAREs). Binding of RA to RAR creates conformational changes that alter the recruitment of nuclear receptor coactivators (NCOAs) or nuclear receptor corepressors (NCORs) leading to a change in the appearance of epigenetic marks on histone H3, i.e., lysine 27 acetylation (K27ac) associated with gene activation or lysine 27 trimethylation (K27me3) associated with gene repression. In the presence of RA, RARE enhancers stimulate recruitment of NCOAs that result in a nearby increase in H3K27ac and/or decrease in H3K27me3 leading to increased target gene expression such as *Meis1* and *Meis2* in embryonic trunk tissue (**B**). In the presence of RA, RARE silencers stimulate recruitment of NCORs that result in a nearby decrease in H3K27ac and/or increase in H3K27me3 leading to decreased target gene expression such as *Fgf8* in embryonic trunk tissue (**C**).

During early development when limb buds are forming, RA is generated by sequential metabolism of retinol (vitamin A) to retinaldehyde by RDH10 [15], followed by the metabolism of retinaldehyde to RA by ALDH1A2 [16]; as *Rdh10* and *Aldh1a2* are expressed in the trunk but not the proximal limb, RA enters the limb by diffusion from the trunk (Figure 1A). RA can be degraded by CYP26 enzymes encoded by *Cyp26a1*, *Cyp26b1*, or *Cyp26c1* [17]; *Cyp26b1* is expressed in distal limb after outgrowth has begun (Figure 1A). RA controls gene expression at the transcriptional level by serving as a ligand for nuclear RA receptors (RARs) that form a heterodimer complex with retinoid X receptor (RXR) when bound to an RA response element (RARE) [18]. Binding of RA induces a conformational shift in RAR which alters binding of coactivators that stimulate deposition of histone H3 K27 acetylation (H3K27ac) marks associated with gene activation, or binding of corepressors that stimulate deposition of histone H3 K27 trimethylation (H3K27me3) marks associated with gene repression [19,20] (Figure 1B,C).

### 2.2. Function of RA during Forelimb Budding

The mechanism through which RA signaling regulates limb development has been controversial as chick limbs treated with RA or RAR antagonists exhibit altered proximodistal patterning [10,11,12], whereas genetic loss of *Aldh1a2* or *Rdh10* in mice does not alter limb proximodistal patterning [8,13,14]. However, genetic loss of *Aldh1a2* or *Rdh10* does disrupt initiation of forelimb development in mice or zebrafish, although hindlimb development is not affected [8,13,15,21,22]. Mouse and zebrafish *Aldh1a2* mutants lack expression of *Tbx5* in trunk lateral plate mesoderm that forms the forelimb field. As *Tbx5* is essential for forelimb initiation [5,6,7] via stimulation of epithelial-to-mesenchymal transition [23] and activation of *Fgf10* [2], RA functions upstream of these important regulators of forelimb bud initiation. Other studies that support a function for RA in forelimb bud initiation include the treatment of chick embryos with the RA synthesis inhibitor disulfiram prior to forelimb bud (wing bud) establishment that prevents forelimb bud initiation [24], plus vitamin A-deficient rat embryos that exhibit forelimb hypoplasia [25] similar to *Rdh10* knockout mice [15]. In addition, in chicken embryos treated with beads containing an RAR antagonist, a phenotype comparable to mouse *Rdh10* mutant stunted forelimbs can be observed that leads to a shorter humerus [26].

**Figure 2:**
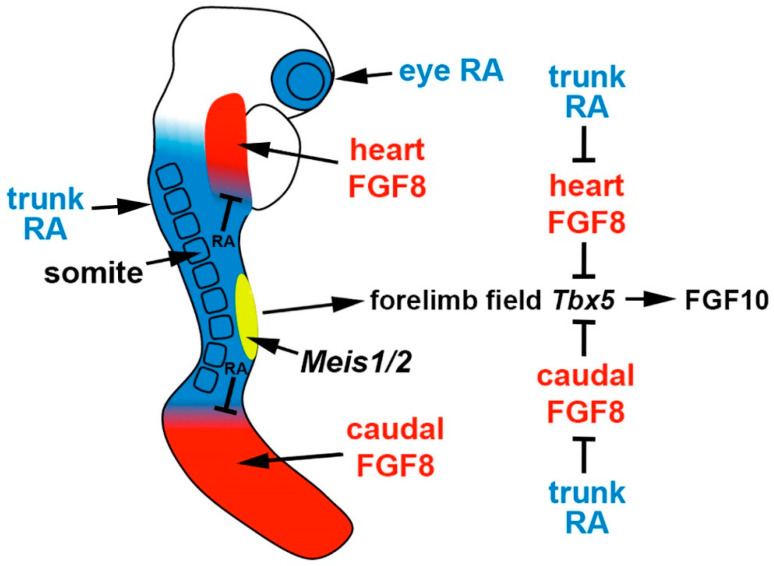
Role of RA signaling, fibroblast growth factor (FGF) signaling, and *Meis1/2* genes in forelimb initiation. In the E8.5 mouse embryo, the forelimb field (yellow) resides within an RA-rich trunk domain (blue) where the RA-generating enzymes encoded by *Rdh10* and *Aldh1a2* are expressed, thus positioned between two domains of FGF8 signaling in the heart and caudal progenitors (red). Trunk RA signaling represses *Fgf8* at the borders of these two domains to permit activation of *Tbx5* in the forelimb field, which then activates FGF10 signaling to stimulate limb outgrowth. Thus, RA acts permissively to activate forelimb *Tbx5* expression. Genetic studies in zebrafish showing that loss of forelimb bud initiation in RA-deficient embryos can be fully rescued by reducing FGF signaling provides evidence that RA is not required to function instructively to activate forelimb *Tbx5* expression. *Meis1/2* genes are required for forelimb bud initiation, but it remains unclear if they activate forelimb *Tbx5* or function in another manner.

Comparison of the forelimb fields of mouse *Aldh1a2* and *Rdh10* mutants revealed that RA activity, normally present throughout the trunk anteroposterior axis, is required to restrict *Fgf8* expression to an anterior trunk domain in the heart and a posterior domain (caudal epiblast or tailbud) on either side of the forelimb field. *Rdh10* mutants exhibit stunted forelimbs and lose early RA activity in the heart and forelimb domains needed to set the boundary of heart *Fgf8* expression, but RA activity still exists caudally which functions to repress caudal *Fgf8* and set its caudal expression boundary; this results in an ectopic *Fgf8* expression domain that stretches posteriorly from the heart into the forelimb field, resulting in a forelimb field *Tbx5* expression domain that is delayed and shortened along the anteroposterior axis [14]. *Aldh1a2*-/- embryos lack RA activity throughout the entire trunk, resulting in ectopic *Fgf8* expression that enters the forelimb field from both the heart and the caudal regions, leading to complete loss of forelimb field *Tbx5* expression and no appearance of forelimb buds [8]. These findings, plus the observation that cultured wild-type mouse embryos treated with FGF8 fail to activate *Tbx5* in the forelimb field [14], demonstrate that the underlying cause of forelimb bud initiation defects in mutants lacking RA synthesis is excessive FGF8 activity in the trunk leading to a disruption of *Tbx5* activation in the forelimb field (Figure 2). This model, in which RA functions permissively to allow forelimb *Tbx5* activation by repressing *Fgf8*, is also supported by the observation that forelimb bud initiation can be rescued in *aldh1a2* mutant zebrafish by introducing a heat-shock-inducible dominant-negative fibroblast growth factor (FGF) receptor [14].

Studies on chick embryos treated with RA combined with mouse enhancer reporter transgene studies suggested that RA signaling may also function instructively to directly activate forelimb *Tbx5* via a potential forelimb enhancer located in intron 2 that contains several HOX-binding sites and a RARE [27,28]; however, this was a degenerate RARE with several base pair mismatches to the RARE consensus sequence [18]. When this potential enhancer (including the degenerate RARE) was deleted in mice using CRISPR/Cas9 gene editing, there was no effect on forelimb bud initiation or later forelimb development [29]. These deletion studies also demonstrated that enhancer reporter transgenes may not always identify endogenous enhancers, indicating that genetic deletion studies are also needed [30]. The challenge of finding limb enhancer regions near *Tbx5* with a functional role in vivo is still ongoing and these studies could allow a better understanding of the underlying mechanism of *Tbx5* activation. With regard to instructive mechanisms, as genetic studies in zebrafish demonstrate that loss of forelimb bud initiation in RA-deficient embryos can be fully rescued by elimination of excess FGF signaling [14], this finding provides evidence that there is no requirement for RA to function instructively to activate forelimb *Tbx5* expression, only a permissive role. Future studies that identify an RA-independent enhancer required in vivo to activate *Tbx5* during forelimb initiation would argue in favor of this hypothesis.

Overall, chick studies relying on treatment with signaling agents such as RA may not reveal normal functions of RA signaling. Similarly, treatment of chick interlimb trunk (between the forelimb and hindlimb buds) with FGF8 stimulates limb bud initiation [31,32], but it is clear to all in the field from mouse *Fgf8* genetic loss-of-function studies that FGF8 is not required for limb bud initiation but instead for limb outgrowth and patterning [3,4]; *Fgf8* is not expressed in trunk lateral plate mesoderm undergoing limb bud initiation and is only expressed in the limb after a substantial bud has already grown.

### 2.3. Mechanism through Which RA Represses Fgf8 in the Developing Trunk

Studies on the mechanism underlying RA repression of trunk *Fgf8* have shown that *Fgf8* is a direct target of RA signaling through an upstream RARE silencer that was shown to be required for repression of caudal *Fgf8* expression in vivo either by transgene analysis or CRISPR/Cas9 deletion of the native RARE [33,34]. ChIP studies on *Aldh1a2* knockout embryos suggest that RA-mediated *Fgf8* repression caudally involves RA-dependent recruitment of nuclear receptor corepressors (NCOR1 and NCOR2), polycomb repressive complex-2 (PRC2) and histone deacetylase-1 (HDAC1) to the *Fgf8* RARE plus nearby deposition of the repressive H3K27me3 mark [33,34]. In addition, RA signaling in trunk cells stimulates movement of the *Fgf8* chromosomal region to the nuclear periphery, which is associated with gene repression [35].

Recently, ChIP-seq (H3K27ac and H3K27me3) analysis combined with RNA-seq analysis performed on wild-type vs. *Aldh1a2-/-* trunk tissue found that the previously known RARE upstream of *Fgf8* is normally marked by H3K27me3, but this mark is lost in the RA-deficient mutant [36]. In this study, two additional RAREs near *Fgf8* were also found to be marked by H3K27me3 when RA is present, highlighting the existence of additional potential *Fgf8* RARE silencers that can be further studied to pursue the mechanism underlying RA repression of *Fgf8* in heart tissues compared to caudal tissues already examined [33,34]. 

### 2.4. Loss of Trunk RA Signaling Does Not Affect Hindlimb Bud Initiation

Hindlimb bud initiation is not perturbed by a loss of trunk RA activity in *Aldh1a2* and *Rdh10* mutants [13,15]. Hindlimb buds do not express *Tbx5,* but instead use *Tbx4* and *Pitx1* to initiate expression of *Fgf10* to stimulate outgrowth [9,37,38]. Expression of *Tbx4* and *Pitx1* is unaffected in the hindlimbs of mouse *Aldh1a2* and *Rdh10* mutants lacking RA activity [8,13].

## 3. Requirement of *Meis* Genes for Forelimb Bud Initiation

After the *Drosophila Meis* homolog was shown to be required for proximodistal patterning of fly limbs [39], subsequent studies on vertebrate *Meis1* and *Meis2* demonstrated that both are expressed throughout the trunk mesoderm (including lateral plate mesoderm that gives rise to limb mesoderm) and in the proximal region of both forelimb and hindlimb buds, suggesting a role in limb proximodistal patterning [10]. Studies on chick limbs showed that treatment with RA or RAR antagonists alters proximal-specific expression of *Meis1/2,* with RA expanding expression into the distal domain, and RAR antagonists eliminating proximal expression [10,11,12]. However, genetic loss of RA signaling in mouse does not alter proximal-specific expression of *Meis1/2* despite loss of all RA signaling activity in the trunk and limb mesoderm [8,13,14]. In addition, no change in *Meis2* expression in the interdigital tissues of the mouse was found in *Rdh10* mutants lacking limb RA signaling [40]. Prior to forelimb bud initiation, RA is also not essential for *Meis1/2* expression in trunk lateral plate mesoderm that gives rise to limb buds, but loss of RA does reduce *Meis1/2* expression in somites [14,36].

In order to globally identify RA target genes in the trunk just prior to forelimb bud initiation, studies were performed that combined epigenetic ChIP-seq (H3K27ac and H3K27me3) with RNA-seq to compare E8.5 wild-type vs *Aldh1a2*-/- trunks lacking RA synthesis. Candidate targets were defined as genes with RA-regulated mRNA abundance that also have nearby RA-regulated H3K27ac (gene activation) or H3K27me3 (gene repression) marks associated with conserved RAREs. This approach identified many previously known RA target genes that control trunk formation, plus additional RA target genes were identified including *Meis1* and *Meis2* [36]. *Meis1/2* were both found to have nearby RAREs that are highly conserved from mammals to reptiles or frogs [36]. Together with previous findings, these new findings suggest that *Meis1/2* are normally activated by RA in the trunk somitic mesoderm but not in the lateral plate mesoderm or limbs, and that the effects of RA and RAR antagonist treatments on *Meis1/2* expression in limb are due to off-target effects in which high concentrations of these reagents target *Meis1/2* RAREs in limb tissue to usurp control of these genes and override their normal limb activators and repressors. 

Genetic studies in mice have been performed to address *Meis1/2* function during limb development. The *Meis1* knockout is lethal at E11.5 due to hematopoietic defects, and the *Meis2* knockout is lethal at E14.5 with defects in cardiac and cranial neural crest, but in both cases no defects in body axis or limb development were observed [41,42]. However, redundancy between *Meis1* and *Meis2* may have masked a defect. CRISPR/Cas9 gene editing was performed to generate *Meis1/2* double mutants in mice, and dissection at E10.5 resulted in embryos that exhibit a body axis defect (small somites) with developmental arrest at E9.5, plus a lack of forelimb buds which are normally easily visible at E9.5; arrested development prevented analysis of hindlimb buds which develop after forelimb buds [36]. Thus, global knockout of *Meis1/2* unexpectedly resulted in the loss of forelimb bud initiation, which indicates an important role in limb development, in addition to a role in somitogenesis (Figure 2).

## 4. *Meis* Genes and FGF Signaling Are Required for Limb Proximodistal Patterning but RA Signaling Is Dispensable

### 4.1. Chick Studies Support a Two-Signal Model for Limb Proximodistal Patterning

Early studies on chick limbs treated with RA suggested a role for RA in limb anteroposterior patterning [43]. However, subsequent studies in chicks provided evidence that RA is not required for limb anteroposterior patterning [44,45], but that sonic hedgehog (SHH) is the diffusible signaling factor required for limb anteroposterior patterning [46]; this requirement for SHH was confirmed in mouse knockout studies [47]. Other studies in chicks demonstrated that treatment of limb buds with RA, RAR antagonists, FGF8, or FGF receptor antagonists alters proximodistal patterning [10,11,12]. Mouse genetic loss-of-function studies verified a requirement for *Fgf8* (and other *Fgf* genes) expressed distally in the apical ectodermal ridge (AER) to control limb proximodistal patterning, including restriction of *Meis1*/*2* expression to the proximal limb [48]. However, loss of RA signaling by knockout of RA-generating enzymes encoded by *Aldh1a2* or *Rdh10* did not result in loss of limb proximodistal patterning or loss of proximal-specific expression of *Meis1*/*2* [8,13,14].

Some researchers supporting the two-signal model raised criticisms, summarized in a review [49], about whether there is a total absence of RA, as measured using the *RARE-lacZ* RA-reporter transgene, in limb mesoderm of E9.5-E10.5 *Aldh1a2* mutants (that need to be treated with a small dose of RA at E7.5 in order to survive to E10.5) or *Rdh10* mutants (that survive to E10.5 without RA treatment). *Aldh1a2* mutants treated with a very small dose of RA at E7.5 were shown to activate *RARE-lacZ* in the trunk mesoderm at E7.5-E8.5 and survive until E10.5, but at E9.5-E10.5 when limbs form, *RARE-lacZ* expression was not detected in the trunk lateral plate mesoderm or limb buds, indicating that the administered RA had been efficiently cleared [8]. In addition, RA titration studies on cultured *Rdh10* mutants that normally have no expression of *RARE*-lacZ in limb or lateral plate mesoderm demonstrated that *RARE-lacZ* expression can be activated by as low as 0.25 nM RA, 100-fold lower than the normal level of limb RA, which is 25 nM, demonstrating that *RARE-lacZ* is a very sensitive RA-reporter [29]. Thus, as RA is reduced by at least 100-fold in limbs and lateral plate mesoderm of *Aldh1a2* and *Rdh10* mutants, it is reasonable to conclude that the concentration would be too low to provide completely normal patterning if RA is required as suggested by the two-signal model.

With regard to a potential interaction of RA and FGF8 during limb patterning, loss of limb RA in *Rdh10* mutants does not alter the expression of *Fgf8* in the AER [13]. This observation demonstrates that although RA does repress *Fgf8* in the body axis to limit its expression to the heart and caudal regions [33,34], one should not assume that RA represses *Fgf8* in other tissues. 

Models based on the treatment of chick embryos with RA and RAR antagonists are weakened by the possibility of off-target effects. As RAR antagonists function as inverse-agonists that silence any gene near an RAR-bound RARE [50], they may dominantly repress genes that have a RARE nearby even though they normally use different regulatory elements for activation in a particular tissue. In the case of *Meis1/2*, the ability of RA and RAR antagonist treatments to effect expression in limb buds, even though loss of endogenous RA does not, can be rationalized by the recent discovery that these genes do require RA for full activation in trunk somites and both genes have functional RAREs [36]. Thus, retinoid treatment regimens may force effects on *Meis1/2* expression in tissues that normally do not use RA to regulate *Meis1/2*.

Despite these conflicting results, a ‘two-signal model’ has been proposed in which RA generated in the trunk diffuses into the proximal region of the limb to promote proximal character by activation of *Meis1/2* expression, with distal FGF8 signaling promoting distal character by repressing *Meis1/2* expression [10,11,12]. This model is also supported by the observation that an RA-degrading enzyme encoded by *Cyp26b1* is expressed in the distal limb under the control of FGF8, with CYP26B1 being required to prevent trunk RA from diffusing into the distal limb, which was reported to ectopically activate *Meis1/2* expression distally [51,52]. In the *Cyp26b1* knockout, *Meis1/2* expression expands into the distal limb, similar to chick RA-treatment studies, but forelimb and hindlimb buds are truncated along the entire proximodistal axis, which is inconsistent with RA functioning to induce proximal identity [51]. In addition, in *Cyp26b1* knockouts, the presence of endogenous RA in distal limbs where it should not normally exist results in a phenotype similar to exogenous RA teratogenesis with increased apoptosis and a block in chondrogenic differentiation, particularly to form the intricate skeletal structures of distal tissues such as hand/foot [53,54] or craniofacial structures [55]. Thus, the presence of endogenous RA in the proximal limb does not necessarily correlate with a designed function in proximodistal patterning of *Meis1/2* expression, but instead may simply indicate diffusion overflow from the trunk where RA is required for body axis formation and forelimb initiation, with this RA being neither necessary nor harmful to proximal limb development. Expression of *Cyp26b1* in the distal limb may not indicate a role in restricting RA signaling and sharpening an RA gradient to set the boundary of *Meis1/2* expression, but may simply function to eliminate RA signaling distally where it is harmful to distal limb development. This would be similar to the function of the related RA-degrading enzyme CYP26A1 in the caudal body axis which functions to remove RA that interferes with body axis extension [56,57], thus restricting RA to the trunk/caudal border where RA is required for spinal cord development (mouse and zebrafish) and somitogenesis (mouse but not zebrafish) [16].

### 4.2. Mouse Genetic Studies Support a One-Signal Model for Limb Proximodistal Patterning 

**Figure 3:**
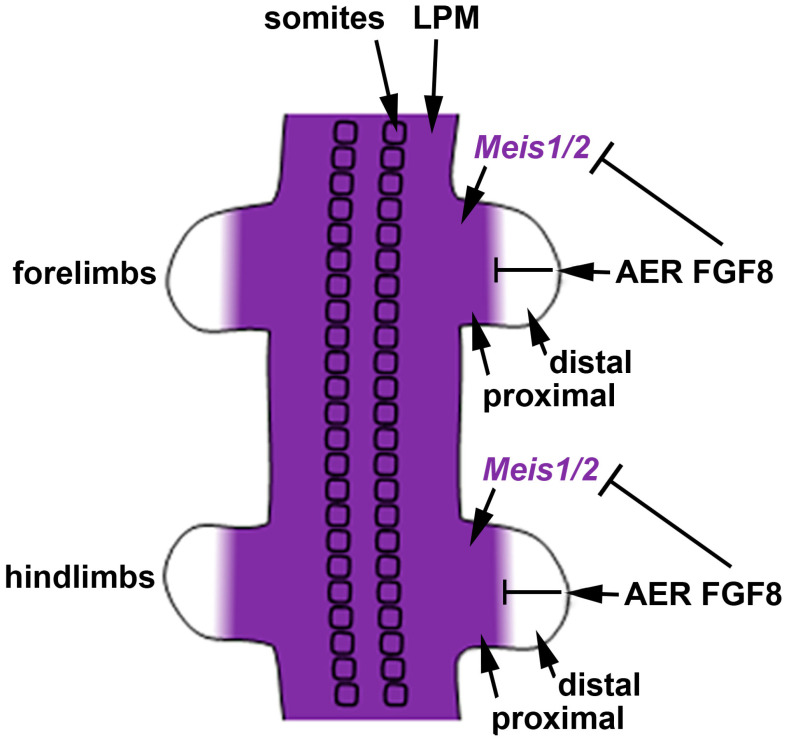
Role of *Meis1/2* genes and FGF signaling in limb proximodistal patterning. In the E10.5 mouse embryo, *Meis1/2* expression that already exists in trunk lateral plate mesoderm (LPM) prior to limb bud initiation extends into the proximal regions of both forelimbs and hindlimbs as they undergo outgrowth from the trunk. *Meis1/2* expression in the trunk LPM and proximal limb does not require RA signaling; the factor(s) that activate *Meis1/2* expression in trunk LPM and limb are unknown. A boundary of *Meis1/2* expression is formed by distal FGF8 (and other FGFs) secreted by the apical ectodermal ridge (AER) that represses *Meis1/2* to limit expression to a proximal limb domain.

We previously suggested that the most parsimonious model for limb proximodistal patterning is a "one-signal model" that is driven by distal FGFs (including FGF8) that stimulate outgrowth, repress *Meis1/2* to generate proximodistal patterning, and activate *Cyp26b1* to remove distal RA from diffusing in from the trunk that would result in limb teratogenesis [18]. Recent studies reported that conditional genetic loss of *Meis1/2* in the mouse limb results in proximodistal patterning defects [58]. Additionally, it was reported that restriction of *Meis1/2* expression to the proximal limb is controlled by the repressive action of distal FGFs (including FGF8) expressed in the AER [58]. As the AER does not form until after the proximal limb is formed, thus delaying production of distal FGFs until after limb bud initiation [32], this results in early *Meis1/2* expression throughout the limb. After the AER is established, *Meis1/2* expression is repressed by distal FGFs, thus creating a boundary of *Meis1/2* expression that leaves it in a proximal position as the distal limb continues to grow free of *Meis1/2* expression (Figure 3). 

## 5. Conclusions and Perspectives

New findings have clarified the mechanisms through which RA signaling, FGF signaling, and *Meis1/2* genes control limb development. We know from recent mouse genetic loss-of-function studies that *Meis1/2* are required for limb bud initiation (at least forelimb) as well as subsequent proximodistal patterning [36,58]. Thus, by combining all genetic studies it can be established that RA signaling functions permissively during forelimb initiation to allow expression of *Tbx5* by repressing trunk *Fgf8* that represses limb field *Tbx5*, but that RA is not required for limb patterning. Furthermore, genetic studies show that RA control of *Meis1/2* occurs in the trunk for somitogenesis but not in limbs, whereas FGF signaling is required for both limb initiation (FGF10) and patterning (FGF8 and other distal FGFs), with distal FGFs restricting *Meis1/2* expression to the proximal limb. With regard to limb patterning, these new genetic findings do not support a two-signal RA-FGF model for proximodistal patterning, but instead support a one-signal model in which a factor other than RA activates *Meis1/2* expression during early limb bud outgrowth followed by action of distal FGFs to repress *Meis1/2*. As *Meis1/2* is expressed in the trunk lateral plate mesoderm prior to limb bud outgrowth from this tissue, and as RA is not essential for *Meis1/2* expression in trunk lateral plate mesoderm, it is possible that whatever factor activates *Meis1/2* in the lateral plate mesoderm continues to allow expression as lateral plate mesodermal cells undergo epithelial-to-mesenchymal transition to form the limb bud. A final proof that RA is not required for *Meis1/2* activation in the proximal limb would come from the identification of such a factor.

Determining the normal functions of RA during development is difficult as pharmacological studies and genetic loss-of-function studies often lead to conflicting results. Similarly, pharmacological studies using treatment with FGF8 led to erroneous conclusions about FGF8 function in limb bud initiation that were later reversed by mouse genetic studies. Thus, a greater reliance on genetic studies will provide clarity on the developmental pathways controlled by RA signaling, FGF signaling, and *Meis1/2*.
